# A Novel Network Pharmacology Strategy to Decode Mechanism of Lang Chuang Wan in Treating Systemic Lupus Erythematosus

**DOI:** 10.3389/fphar.2020.512877

**Published:** 2020-10-02

**Authors:** Yao Gao, Ke-xin Wang, Peng Wang, Xiao Li, Jing-jing Chen, Bo-ya Zhou, Jun-sheng Tian, Dao-gang Guan, Xue-mei Qin, Ai-ping Lu

**Affiliations:** ^1^ Modern Research Center for Traditional Chinese Medicine, Shanxi University, Taiyuan, China; ^2^ Institute of Integrated Bioinformedicine and Translational Science, Hong Kong Baptist University, Hong Kong, Hong Kong; ^3^ Zhijiang College, Zhejiang University of Technology, Shaoxing, China; ^4^ Department of Ultrasound, Eighth Affiliated Hospital of Sun Yat-sen University, Shenzhen, China; ^5^ Department of Biochemistry and Molecular Biology, School of Basic Medical Sciences, Southern Medical University, Guangzhou, China; ^6^ Guangdong Provincial Key Laboratory of Single Cell Technology and Application, Southern Medical University, Guangzhou, China

**Keywords:** Lang Chuang Wan, systemic lupus erythematosus, network pharmacology, optimization space, effective proteins, contribution index

## Abstract

Complex disease is a cascade process which is associated with functional abnormalities in multiple proteins and protein-protein interaction (PPI) networks. One drug one target has not been able to perfectly intervene complex diseases. Increasing evidences show that Chinese herb formula usually treats complex diseases in the form of multi-components and multi-targets. The key step to elucidate the underlying mechanism of formula in traditional Chinese medicine (TCM) is to optimize and capture the important components in the formula. At present, there are several formula optimization models based on network pharmacology has been proposed. Most of these models focus on the 2D/3D similarity of chemical structure of drug components and ignore the functional optimization space based on relationship between pathogenetic genes and drug targets. How to select the key group of effective components (KGEC) from the formula of TCM based on the optimal space which link pathogenic genes and drug targets is a bottleneck problem in network pharmacology. To address this issue, we designed a novel network pharmacological model, which takes Lang Chuang Wan (LCW) treatment of systemic lupus erythematosus (SLE) as the case. We used the weighted gene regulatory network and active components targets network to construct disease-targets-components network, after filtering through the network attribute degree, the optimization space and effective proteins were obtained. And then the KGEC was selected by using contribution index (CI) model based on knapsack algorithm. The results show that the enriched pathways of effective proteins we selected can cover 96% of the pathogenetic genes enriched pathways. After reverse analysis of effective proteins and optimization with CI index model, KGEC with 82 components were obtained, and 105 enriched pathways of KGEC targets were consistent with enriched pathways of pathogenic genes (80.15%). Finally, the key components in KGEC of LCW were evaluated by *in vitro* experiments. These results indicate that the proposed model with good accuracy in screening the KGEC in the formula of TCM, which provides reference for the optimization and mechanism analysis of the formula in TCM.

## Introduction

The key group of effective components (KGEC) in a formula of traditional Chinese medicine (TCM) refer to the pharmacologically active components that are closely related to the positive response to the therapy of the diseases. How to determine the KGEC that play leading roles in the treatment of specific disease is a difficult problem due to the high complexity of the chemical composition and the incompletely understanding the complex multi-targets mechanism of TCM. Selecting the KGEC in the formula of TCM is an important direction in the reduction of non-active components and analysis of the treatment mechanism of formula. At present, there are several formula optimization models based on network pharmacology have been proposed. Most of these models focus on the 2D/3D similarity of chemical structure of drug or components, and ignore the optimized functional space, which couldrepresent effective relationships between drug targets and pathogenetic genes (Wang et al., 2018; Xie et al., 2018; Duan et al., 2019). Increasing evidences confirm that the monomer component of TCM exerts important pharmacological effects through the protein-protein interactions (PPI) (Chen and Cui, 2017; Gan et al., 2018; Guo et al., 2019). Thus, it is desirable to design a module to detect the KGEC and infer the potential mechanisms of formula on complex disease based on chemical properties analysis, targets prediction, and construction of functional optimization space.

Systemic Lupus Erythematosus (SLE) is an autoimmune disease involving multiple systems and organs, with complicated clinical manifestations and persistent. Most of them are found in young women, and the incidence ratio of male to female is 1: 5 ~ 10 (Veeranki and Choubey, 2010; Dorner and Furie, 2019). Previous researches indicate that the SLE may be related to heredity, immune disorder, endocrine abnormality and environmental factors. Currently, western medicine mainly adopts non-steroidal anti-inflammatory drugs, antimalarial drugs, glucocorticoids, immunosuppressive agents, plasma treatment, and systemic lymph node irradiation therapy for the treatment of SLE (Loram et al., 2015; Wallace, 2015). However, so far these drugs and methods can only temporarily control the disease. At the same time, some toxic and side effects of western medicine are becoming increasingly apparent. Intervention therapy of TCM can not only effectively relieve clinical symptoms, but also reduce the toxic and side effects of western medicine (Huang et al., 2016; Ma et al., 2016). Increasing evidences proved that TCM play important roles in both acute and remission phases of SLE.

In recent years, TCM has been widely used in the treatment of SLE and has achieved remarkable results. With the continuous improvement of its therapeutic effect, it has gradually attracted the attention of medical experts and scholars at home and abroad (Ma et al., 2016). At present, the TCM formula, Lang Chuang Wan (LCW) is widely used in the treatment of SLE with TCM. LCW generally comprised of 16 herbs: *Lonicera japonica* Thunb. (Jinyinhua, 53.6 g), *Forsythia suspensa* (Thunb.) Vahl (Lianqiao, 53.6 g), *Taraxacum mongolicum* Hand. (Pugongying, 53.6 g), *Coptis chinensis* Franch. (Huanglian, 13.4 g), *Rehjnannia glutinosa* Libosch. (Dihuang, 53.6 g), *Rheum officinale* Baill. (Dahuang, 20.1 g), *Glycyrrhiza uralensis* Fisch. (Gancao, 13.4 g), *Scolopendra subspinipes mutilans* L. Koch (Wugong, 2.42 g), *Paeonia ladiflora* Pall. (Chishao, 26.8 g), *Angelica sinensis* (Oliv.) Diels (Danggui, 13.4 g), *Salvia miltiorrhiza* Bge. (Danshen, 13.4 g), *Scrophularia ningpoensis* Hemsl. (Xuanshen, 53.6 g), *Prunus persica* (L.) Batsch (Taoren, 26.8 g), *Carthamus tinctorius* L. (Honghua, 20.1 g), *Cryptotympana pustulata* Fabricius (Chantui, 53.6 g), and *Fritillaria thunbergii* Miq. (Zhebeimu, 26.8 g). In this Chinese formula, Flos Lonicerae, Fructus Forsythiae and Herba Taraxaci have the function of clearing heat-toxin, eliminating carbuncles, and resolving masses, Coptidis Rhizoma has the function of clearing heart fire, the Rehmanniae Radix mainly focus on cooling blood, nourishing yin and promoting fluid production, Radix et Rhizoma Rhei play roles in clearing heat cooling blood promoting blood circulation; Glycyrrhrizae Radix used for clearing heat-toxin harmonizing various drugs; Radix Paeoniae Rubra, Saviae Miltiorrhizae Radix and Carthami Flos usually used for clearing heat cooling blood; Scolopendra used for removing toxic substance resolving masses, detumescence, and relieving pain; Radix Angelicae Sinensis used for nourishing and activating blood; Radix Scrophulariae used for clearing heat-toxin nourishing yin for lowering fire; Semen Persicae used for promoting blood circulation removing blood stasis; Periostracum Cicadae used for clearing heat; Bulbus Fritillariae Thunbergii used for clearing heat dissipating phlegm and resolving masses. The whole recipe has the functions of clearing away heat and toxic materials, cooling blood and promoting blood circulation. In pharmacologic studies, LCW may has the effect of inhibiting humoral immune function and enhancing cellular immune function. Additionally, LCW also obvious inhibitory effect on acute and chronic inflammation and allergy in rats, and can promote fibrinolytic activity. These studies confirmed that the LCW could be beneficial in the treatment of patients with SLE at comprehensive level. Nevertheless, not any document expounds the key components and underlying therapeutic mechanism of LCW for clinically benefit to SLE (Wang, 1989; Wang et al., 1991).

Currently, a novel network pharmacology module was designed to detect the KGEC and elucidate the therapeutic mechanisms of LCW in the treatment of SLE. During this process, the weighted gene regulatory network of SLE disease was constructed and used for constructing of optimization space. All LCW components were collected from databases and literature. The potential active components were selected from all components based on published ADME-related models; the targets of these active components were predicted by three published prediction tools. The active components and their targets were used for building the components-targets (C-T) network. The weighted gene regulatory network and C-T networks were used to construct optimization space to determine the effective proteins. The effective proteins selected from optimization space were used to screen the effective components. The contribution index (CI) module was employed to optimize effective components and get the KGEC, which would be used to illustrate the molecular mechanism of LCW in the therapy of SLE. Finally, the key components in KGEC of LCW were evaluated by *in vitro* experiments.

## Methods

### Construct Weighted Gene Regulatory Network of SLE

In order to construct comprehensive weight gene network of SLE, the PPI data were derived from public databases BioGRID, STRING, Dip, HPRD, Intact, Mint and Reactome (Guan et al., 2014). Genes from DisGeNET (Pinero et al., 2017) were reported to be related to SLE were extracted and mapped to the PPI network to construct the weighted gene regulatory network of SLE. Cytoscape (Version 3.5.1) was utilized to visualize the network.

### LCW Content Determination

#### Reagents and Chemicals

High performance liquid chromatography (HPLC)-grade acetonitrile and HPLC grade formic acid were obtained from Thermo-Fisher (USA). Reference standards provided by the National Institute on Drug Abuse of China:Hydroxysafflor-Yellow-A (batch number: 111637-200502), amygdalin (batch number: 110820-200403), paeoniflorin (batch number: 0905-9805), caffeic acid (batch number: 110728-200506), phillyrin (batch number: 120908-200914), liquiritin (batch number:111610-200503), peimisine (batch number: 0750-9303), harpagoside (batch number: 712-9403), rhein (batch number: 0902-200207), Z-Ligustilide (batch number: 927-9908), berberine (batch number: 0713-200107), tanshinone II a (batch number: 0766-200011), catalpol (batch number: 0808-9602). The purities of all standards were no less than 98% and suitable for liquid chromatography-tandem mass spectrometry (LC-MS/MS) analysis. LCW was purchased from Changchun Overseas Pharmaceutical Co. Ltd.

#### Preparation of Samples and Standard Solution

LCW (batch number: 20190601) was grinded into powder. Each sample of LCW powder (0.5 g) was weighed precisely and ultrasonically extracted in 50 ml hydrochloric-acid methanol (1:100) for 30 min. Supplement the weight lost with hydrochloric acid-methanol (1:100) solution and then filtered through 0.22 μm nylon membrane filters. The filtrate was analyzed directly by UPLC-ESI-MS/MS. At the same time, a stock solution containing 13 reference standards was prepared in methanol. All solutions were stored at 4°C prior to analysis.

#### Instrument and UPLC-ESI-MS/MS Conditions

Chemical profiling of LCW and reference standards were detected by an Agilent ultra-performance liquid chromatography system (UHPLC) (Agilent, USA) coupled to a Q-trap 3200 (AB SCIEX, USA). Chromatography separation was carried out on a Waters ACQUITYUPLC HSS T3 column (2.1 mm × 100 mm, 1.8 μm) maintained at 40°C. The mobile phase consisted of 0.1% formic acid in water (A) and 0.1% formic acid in acetonitrile (B), and run under the following program: 0 ~ 1 min, 15% B; 1 ~ 4 min, 15 ~ 45% B; 4 ~ 10 min, 45 ~ 60% B, 10 ~ 15 min, 60 ~ 90% B; 15 ~ 18 min, 90 ~ 95% B. The sample injection volume was 5 μl and the flow rate was set at 0.2 ml/min. The mass spectrometer was fitted with an electrospray ionization source and the mass detection was operated in both positive and negative ion modes with the following setting: ion source temperature, 500°C; Sheath gas velocity, 50 psi; Auxiliary gas flow rate, 12 L/min; scan range, m/z 150–900.

### Collect and Select Chemical Components of LCW

All components of LCW were collected from four published natural product data sources: TCMSP database (Ru et al., 2014), Traditional Chinese Medicine integrated database (Xue et al., 2013), Traditional Chinese Medicine database@Taiwan (Chen, 2011), and YaTCM (Li et al., 2018). For all components, the initial structure formats (e.g., mol2 and SDF) were transformed into unified SDF format using Open Babel toolkit (version 2.4.1). Subsequently, the properties of components were retrieved from TCMSP, including molecular weight (MW), oral bioavailability (OB), Caco-2 permeability (Caco-2), and DL (drug-likeness).

Three ADME-related models, including OB, Caco-2, and DL, were employed to screen the bioactive molecules. OB (%F) represents the percentage of an orally administered dose of unchanged drug that reaches the systemic circulation, which reveals the convergence of the ADME process (Xu et al., 2012). High oral bioavailability is often a key indicator to determine the drug-like property of bioactive molecules as therapeutic agents. The components with suitable OB≥30% were chosen as candidate components for further research. Human intestinal cell line Caco-2 is used as an efficient *in vitro* model to study the passive diffusion of drugs across the intestinal epithelium, the ingredients’ transport rates (nm/s) in Caco-2 monolayers represent the intestinal epithelial permeability in TCMSP. Components with Caco-2>-0.4 were chosen as the candidate components, because the components with a Caco-2 value less than -0.4 were not permeable. Drug-likeness is a qualitative concept used in drug design for an estimate on how “drug-like” a prospective compound is, which helps to optimize pharmacokinetic and pharmaceutical properties, such as solubility and chemical stability. The “drug-like” level of the components is 0.18, which is used as a selection criterion for the “drug-like” components in the traditional Chinese herbs (Tao et al., 2013). After ADME screening, some components that did not meet the three screening criteria were also selected because of their high content and high biological activity. These will be used in conjunction with ADME screening as a follow-up study.

### Predict Targets of Active Components

To obtain the targets of active components in LCW, the commonly used prediction tools, i.e., Similarity Ensemble Approach (SEA) (Tao et al., 2013), HitPick (Liu X. et al., 2013), and Swiss Target Prediction (Gfeller et al., 2014), were employed to identify the targets. All chemical structures were prepared and converted into canonical SMILES using Open Babel toolkit (version 2.4.1).

### Define the Optimization Space and Evaluate the Effective Proteins

Construction of the optimization space is able to maximize the search for targets of small molecules that are highly relevant to pathogenetic genes. Firstly, we used the weighted gene regulatory network and active components targets network to construct the disease-targets-components network. Degree is an important topological property in the network that can be used to evaluate the importance of nodes in the network. The nodes with higher degree than the average degree of all nodes in the disease-targets-components network were identified as hub nodes. Following this rule, the passed nodes and their edges in the disease-targets-components network were kept and defined as optimization space. The whole process can be described as follows:

We defined Net*_ppi_* = {*N*, *E*}, *N* means nodes that represent proteins, *E* means edges that represent protein–protein interactions derived from public databases BioGRID, STRING, Dip, HPRD, Intact, Mint, and Reactome. *T_tar_* = {*P*_1_ *_tar_*, *P*_2_ *_tar_*… … *P_ntar_*} means the predicted targets of active components. *D_dis_* = {*G*_1_ *_dis_*, *G*_2_ *_dis_*… … *G_ndis_*} means the pathogenic genes of SLE. The optimization space can be calculated by the following steps.

$$L_{tar}=\left\{P_{1tar},P_{2tar}......P_{ntar}\right\}$$

$$L_{dis}=\left\{P_{1dis},P_{2dis}......P_{ndis}\right\}$$

$$Net_{tarppi}=\left\{N_{tar},E_{tar}\right\}=Net_{ppi}\cap L_{tar}\;,\;Net_{tar}\in N;E_{tar}\in E$$

$$Net_{disppi}=\left\{N_{dis},E_{dis}\right\}=Net_{ppi}\cap L_{dis}\;,\;Net_{dis}\in N;E_{dis}\in E$$

$$Net_{tar-disppi}=Net_{ppi}\cap Net_{disppi}\cap Net_{tarppi}$$

$$D_{avg}=\left(\sum_{i=1}^{k}d_{i}\right)/k$$

$$OptS=\cup_{i=1}^{k}d_{\left(Net_{tar-disppi}\right)i}>D_{avg}$$

Where $Net_{tar^{ppi}}$ is the network of predicted targets, the $Net_{dis^{ppi}}$ is the network of pathogenic genes.$Net_{tar-dis^{ppi}}$ represents the disease-targets-components network. *D_avg_* is the average degree of all nodes in the disease-targets-components network. *k* means the number of nodes in the disease-targets-components network. OptS represent the optimization space. The nodes in the optimization space were identified as effective proteins.

### Develop a CI Model to Select KGEC

To optimize effective components and get the KGEC, which would be used to illustrate the molecular mechanism of LCW in the therapy of SLE. Active components which are associated with effective proteins were extracted as λ = {λ_1_, λ_2_, λ_3_… … λ*_m_*}, The target number of each active compounds be defined as ω = {ω_1_, ω_2_, ω_3_… …ω*_m_*}, then coverage of the target number for each active compounds defined as *ν* = {v_1_, v_2_, v_3_… … v*_m_*}, the dynamic-0-1 knapsack algorithm can be described as:

Input: *ν* and ω, the number of active components m and the number of effective *ν* proteins W.

Output: The optimal KGEC.

$$\begin{array}{l} for\;\omega \leftarrow 0\;to\;W\;do\\ \;c[0,\omega ]\leftarrow 0\\ end\;for\\ fori\leftarrow 0\;\;to\;m\;do\\ \;c[i,0\;]\leftarrow 0\\ \;for\;\omega \leftarrow 1\;\;to\;W\;do\\ \;if\;\omega_{i}\ll \omega \;then\\ \;if\;v_{i}+c[i-1,\;\omega -\omega_{i}]>c[i-1,\omega ]\;then\\ \;c[i,\omega ]\leftarrow v_{i}+c[i-1,\;\omega -\omega_{i}]\\ \;else\\ \;c[i,\omega ]\leftarrow c[i-1,\omega ]\\ \;end\;if\\ \;else\\ \;c[i,\omega ]\leftarrow c[i-1,\omega ]\\ \;end\;if\\ \;end\;for\\ end\;for\\ return\;CI=c[k,W] \end{array}$$

### Gene Ontology and Pathway Analysis

To analyze the main function of the targets, the clusterProfiler package of R software was used to perform Gene Ontology (GO) analysis. *p*-values were set at 0.05 as the cut-off criterion. The clusterProfiler package of R software (Yu et al., 2012) was employed to classify the biological terms and to analyze the gene cluster enrichment automatically. The latest pathway data were obtained from the Kyoto Encyclopedia of Genes and Genomes (KEGG) database (Draghici et al., 2007) for KEGG pathway enrichment analyses. *p*-values were set at 0.05 as the cut-off criterion. The ggplot2 package was used to create graphs in R statistical programming language (version 3.4.2). The results of analysis were annotated by Pathview (Luo et al., 2017) in the R Bioconductor package (https://www.bioconductor.org/).

### Experimental Validation

#### Chemicals and Reagents

Liquiritin and ferulic acid (≧98% purity by HPLC, PUYI BIOLOGY; Jangsu, China). Resiquimod (≧99.6%, Topscience Co., Ltd. China). Fetal bovine serum (FBS) (Sangon Biotech (Shanghai) Co., Ltd., China). RPMI-1640 (Sangon Biotech (Shanghai) Co., Ltd., China). β-actin, Phospho-ERK1/2, Phospho-AKT, and Phospho-PI3K (CST, USA).

#### Cell Culture and Drug Treatment

RAW 264.7 cells (Cell Bank of Chinese Academy of Sciences, China) were cultured in DMEM-H with 10% FBS at 37°C, in an atmosphere containing 5% CO_2,_ humidified 95%. RAW 264.7 cells were seeded on 96-well plates with 1×10^4^ per/well, 100 mm dishes with 1×10^6^ per/dishes, and cultured for 24 h. After incubation, the RAW 264.7 cells were co-incubated with different concentrations of liquiritin, ferulic acid, and resiquimod for 24 h. The concentration of resiquimod was selected at 0.1 μg/ml.

#### Cell Viability Assay

RAW 264.7 cells were seeded in 96-well plates. After drug treatment, the culture medium was removed, 100 μl 0.5 mg/ml MTT (Sangon Biotech (Shanghai) Co., Ltd., China) solution was added. After 4 h, the culture medium was removed and 100 μl DMSO (Sangon Biotech (Shanghai) Co., Ltd., China) was added. The absorbance at 570 nm was measured with a microplate reader (BioTek Epoch, USA). Cell viability is expressed as a percentage of the control.

#### Measurement of IL-6 Levels

The cells were cultured in 6-well plates. After relevant treatment, the cells were collected and centrifuged to obtain a cell pellet and supernatant. The cell pellet and supernatant were stored at −80°C until required for analysis. The level of IL-6 was determined by commercial assay kits (Nanjing Jiancheng, China).

#### Western Blot Analyses

RAW 264.7 cells were seeded in 100 mm dishes. At the end of the treatments, the cells were harvested and washed twice with cold PBS. The cells were lysed with RIPA lysis buffer (Beyotime, China) containing 1% phenylmethylsulfonylfluoride (PMSF, Beyotime, China). The whole-cell lysates were centrifuged at 12,000 rpm/min for 15 min at 4°C, and the supernatants were collected. Protein concentrations were determined by bicinchoninic acid assay. Equal amounts of protein (50 μg) were separated by electrophoresis on 12% sodium dodecyl sulphate polyacrylamide gels and transferred onto PVDF membranes. These membranes were incubated with 5% (w/v) non-fat milk powder in Tris-buffered saline containing 0.1% (v/v) Tween-20 (TBST) for 2 h to block nonspecific binding sites. The membranes were then incubated overnight at 4°C with the primary antibodies. After washing with TBST, the membranes were incubated for 2 h at room temperature with the fluorescent secondary antibodies. After rewashing with TBST, the membranes were scanning by a fluorescent scanner (Odclyssey CLX, Gene company limi ed, USA).

## Results

**Figure 1:**
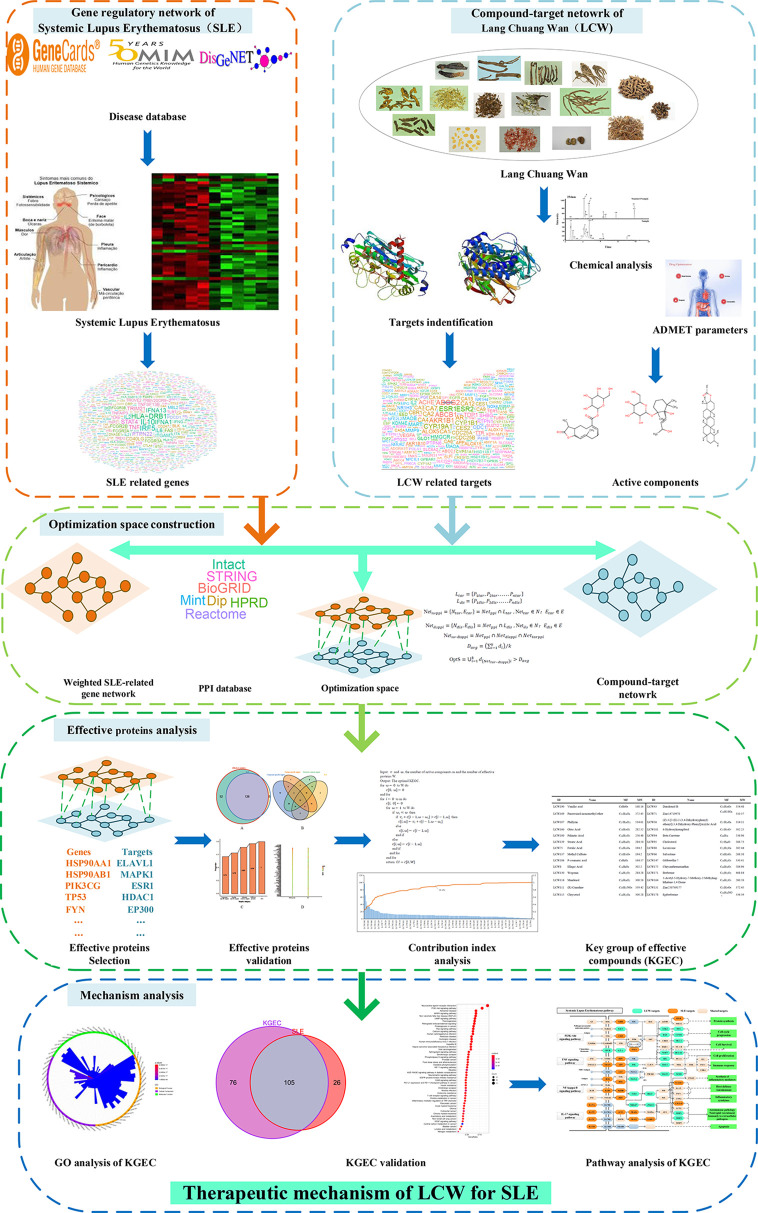
The flowchart of our proposed network pharmacology approach. Experimental methods include gene regulatory network of SLE, compound-target netowork of LCW, optimization space construction, effective proteins analysis and mechanism analysis. SLE represents systemic lupus erythematosus, LCW represents Lang Chuang Wan, KGEC represents key group of effective components.

In this report, a novel network pharmacology module was designed to detect the KGEC and elucidate the therapeutic mechanisms of LCW in the treatment of SLE (**Figure 1**). Firstly, all LCW components were collected from databases and literature. Next, the potential active components were selected from all LCW components based on published ADME-related models. The targets of these active components were predicted by three online prediction tools. Then the weighted gene regulatory network and active components targets network were used to construct optimization space for determining the effective proteins. The effective proteins were used to select the KGEC based on CI module and then the KGEC was used to infer the underlying molecular mechanism of LCW in treating SLE. Finally, the key components in KGEC of LCW were evaluated by *in vitro* experiments.

### Construct Weighted Gene Regulatory Network of SLE

**Figure 2:**
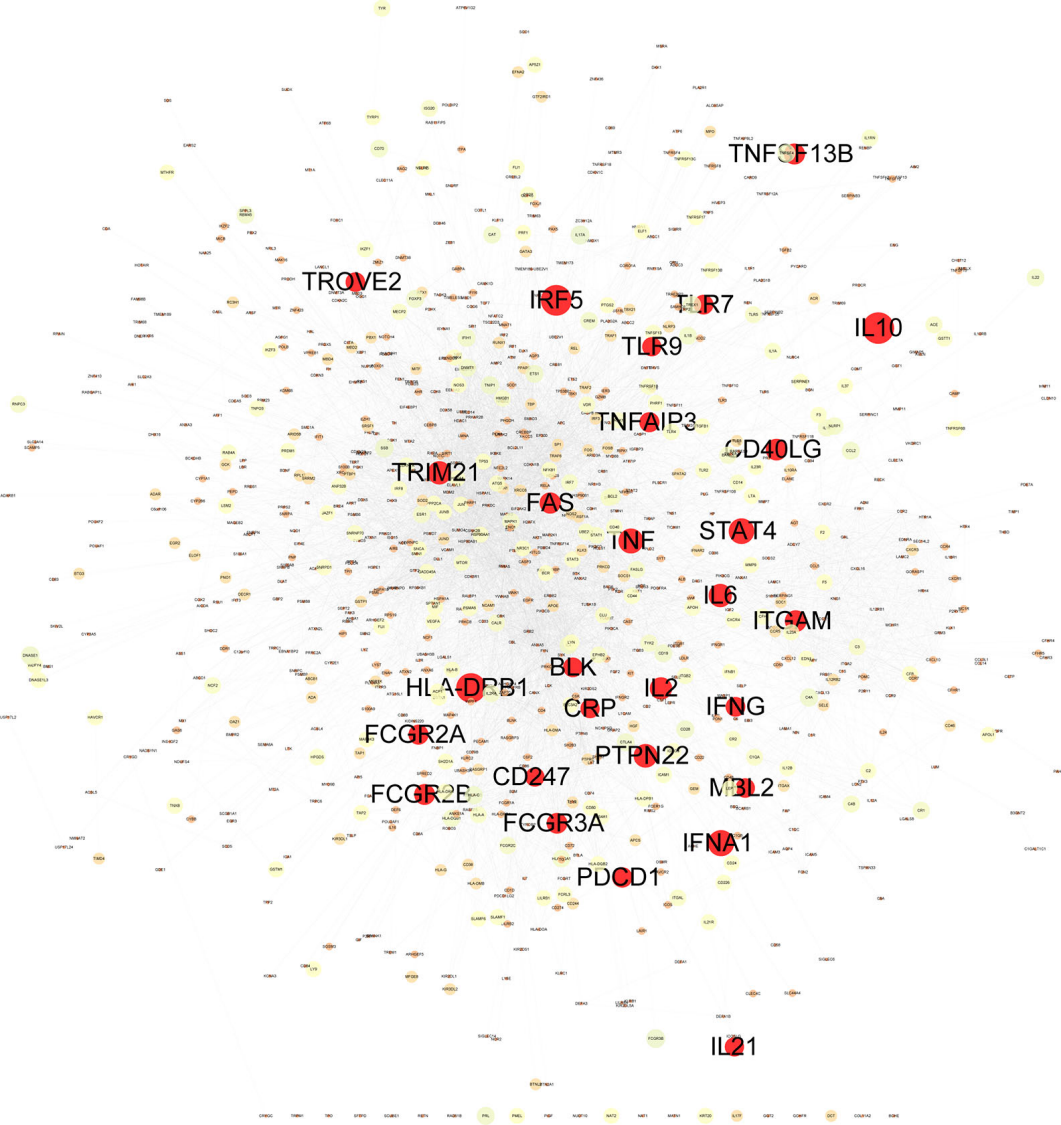
Weighted gene regulatory network of SLE. The weighted gene regulatory network contains 950 nodes and 6,984 edges. The number of literature reports of one node represent the weight of the node. The red nodes list the top 30 of SLE pathogenetic genes in the weighted gene regulatory network.

**Figure 3:**
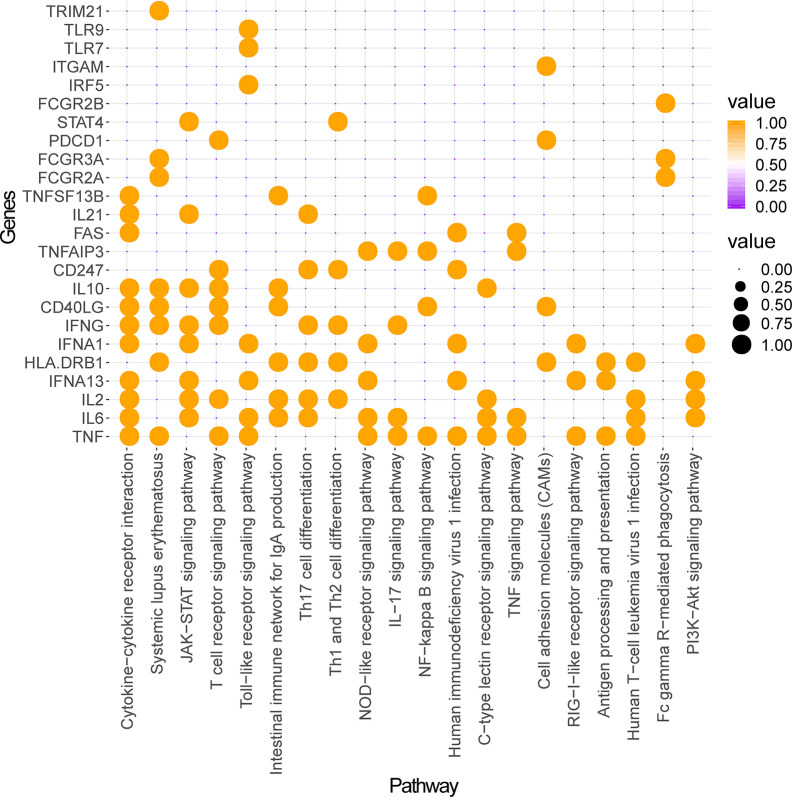
Pathway enrichment analysis of top 30 weighted genes of SLE. The ordinate represents genes and the abscissa represents enriched pathways. Orange nodes represent genes enriched in a given pathway and no nodes represent genes not enriched in this pathway.

Construction and analysis of weighted gene regulatory network is the basis and key step to understand the pathogenesis and provide intervention strategies of SLE. In order to construct a comprehensive weighted gene network of SLE, the PPI data sets from public databases BioGRID, STRING, Dip, HPRD, Intact, Mint and Reactome were used to construct the PPI network. 1201 genes from DisGeNET which confirmed associated with SLE were extracted and mapped to the PPI network to construct the weighted gene regulatory network of SLE. The weighted gene regulatory network contains 950 nodes and 6,984 edges (**Figure 2**). The number of literature reports of one node represent the weight of the node (**Table S1**). Eight out of top 30 genes with the highest weight in the gene regulatory network enriched in the common SLE pathways (hsa05322), including TNF (Geng et al., 2014), HLA-DRB1 (Shimane et al., 2013), IFNG (Leng et al., 2016), CD40LG (Wu et al., 2016), IL10 (Liu P. et al., 2013), FCGR3A (Kyogoku et al., 2013), FCGR2A (Bentham et al., 2015) and TRIM21 (Kyriakidis et al., 2014). These genes are also enriched in cytokine-cytokine receptor interaction, T cell receptor signaling pathway and Th17 cell differentiation, which are closely related to SLE (**Figure 3**). These results indicate that the weight gene regulatory network and weight genes can reflect the pathogenesis of SLE, which also provides a reliable reference for the next step to construct the optimization space.

### Chemical Components Analysis

**Figure 4:**
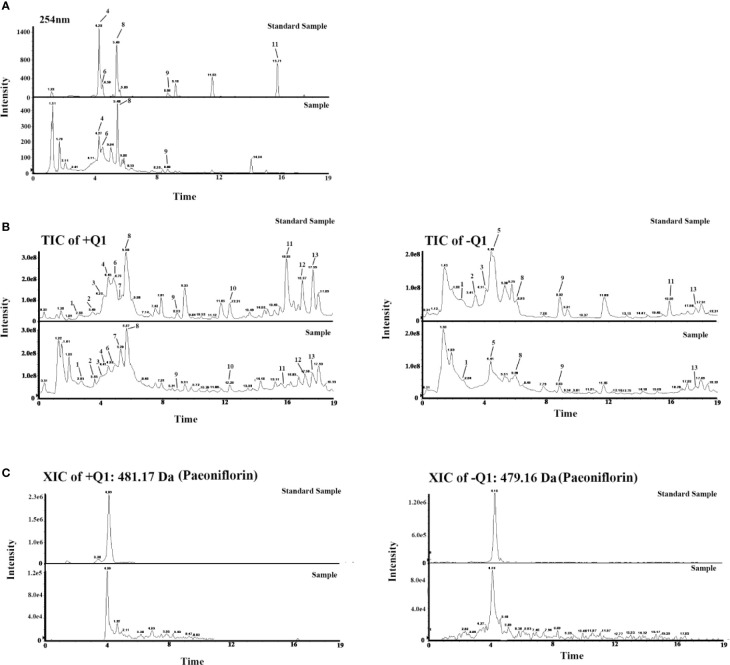
**(A)** Chromatograms of LCW and standard samples. **(B)** Total ion Current (TIC) chromatograms of LCW and standards samples. **(C)** Extracted ion current (XIC) chromatograms of paeoniflorin in LCW and standard samples. 1) Hydroxysafflor-Yellow-A, 2) Amygdalin, 3) Paeoniflorin, 4) Caffeic Acid, 5) Phillyrin, 6) Liquiritin, 7) Peimisine, 8) Harpagoside, 9) Rhein, 10) Z-Ligustilide, 11) Berberine, 12) Tanshinone II a, and 13) Catalpol.

Thirteen components in the chromatograms of the LCW sample were identified and assigned by comparing the retention time with those of the reference compounds (**Figure 4**). The 13 components are hydroxysafflor-Yellow-A, amygdalin, paeoniflorin, caffeic acid, phillyrin, liquiritin, peimisine, harpagoside, rhein, Z-Ligustilide, berberine, tanshinone II a, and catalpol (**Table 1**). The pharmacopoeia defines that the content of berberine should not be less than 0.4 mg/g, and we get the content of berberine as 0.57 mg/g by chemical analysis. These results confirmed that the content of berberine in LCW meets the requirements of pharmacopoeia. Chemical Components analysis provides a reference for the screening of active components in LCW for further analysis.

**Table 1:** Information of chemical components in LCW.

No	TR	Name	Formula	Molecular Weight	m/z	ion	Area (Standard)	Area (LCW)	Content (mg)
1	2.56	Hydroxysafflor-Yellow-A	C_27_H_32_O_16_	612.17	613.18	M+H	1.06E+07	7.19E+05	0.34
2	3.41	Amygdalin	C_20_H_27_NO_11_	457.16	458.17	M+H	1.36E+06	8.70E+04	0.32
3	4.1	Paeoniflorin	C_23_H_28_O_11_	480.16	481.17	M+H	2.79E+07	3.15E+05	0.06
4	4.42	Caffeic Acid	C_9_H_8_O_4_	180.04	181.05	M+H	3.75E+06	1.14E+06	1.52
5	4.48	Phillyrin	C_29_H_36_O_15_	624.20	623.19	M-H	9.24E+07	3.57E+06	0.19
6	4.7	Liquiritin	C_21_H_22_O_9_	418.13	419.14	M+H	2.40E+06	4.30E+05	0.89
7	5.1	Peimisine	C_27_H_45_NO_3_	431.34	430.33	M-H	6.98E+05	5.28E+04	0.38
8	6.08	Harpagoside	C_24_H_30_O_11_	494.18	493.17	M-H	2.31E+07	2.11E+06	0.46
9	8.81	Rhein	C_15_H_8_O_6_	284.03	285.04	M+H	1.07E+07	3.30E+06	1.54
10	12.19	Z-Ligustilide	C_12_H_14_O_2_	190.10	191.11	M-H	5.77E+06	2.24E+06	1.94
11	15.89	Berberine	C_17_H_17_N	235.14	236.15	M-H	5.10E+06	5.84E+05	0.57
12	16.87	Tanshinone II a	C_19_H_18_O_3_	294.13	295.14	M+H	1.61E+08	3.94E+06	0.12
13	17.57	Catalpol	C_15_H_22_O_10_	362.12	363.13	M+H	7.06E+06	3.35E+05	0.24

### Select Potential Active Components

By a systematic search of components from public databases of 16 herbs, a total of 1693 components were retrieved in LCW in **Table 2**. The detail information of these components was provided in **Table S2**. TCM formula usually contains multiple components, only a few components have satisfactory pharmacodynamic and pharmacokinetic properties. In this study, three ADME-related models, OB, Caco-2, and DL were employed to screen the active components. The components with OB values higher than 30%, Caco-2 values higher than -0.4 and DL values higher than 0.18 were retained for further investigation. After ADME screening, some components that did not meet the three screening criteria were selected because of their high content and high biological activity, which has been reported in the literature and our UPLC-ESI-MS/MS analysis. Finally, 193 active components were filtered out of the 1,693 components of LCW. The detail information was shown in **Table S3**.

**Table 2:** The number of LCW components collected in the published databases.

Herbs	components	Herbs	components
*Lonicera japonica* Thunb. (JYH)	239	*Paeonia ladiflora* Pall. (CS)	75
*Forsythia suspensa* (Thunb.) Vahl (LQ)	153	*Angelica sinensis* (Oliv.) Diels (DG)	126
*Taraxacum mongolicum* Hand. (PGY)	77	*Salvia miltiorrhiza* Bge. (DS)	203
*Coptis chinensis* Franch. (HL)	33	*Scrophularia ningpoensis* Hemsl. (XS)	47
*Rehjnannia glutinosa* Libosch. (DH)	76	*Prunus persica* (L.) Batsch (TR)	66
*Rheum officinale* Baill. (DH)	92	*Carthamus tinctorius* L. (HH)	190
*Glycyrrhiza uralensis* Fisch. (GC)	280	*Cryptotympana pustulata* Fabricius (CT)	8
*Scolopendra subspinipes mutilans* L.	8	*Fritillaria thunbergii* Miq. (ZBM)	17
Koch (WG)			
Total		1693	

### Shared Components of Herbs in LCW

As can be seen from **Table S2**, there exist more than 20 active components shared by two or more herbs in LCW. For example, beta-sitosterol (LCW5), a common component of 10 herbs such as CS, DH, DG, HH, JYH, LQ, TR, XS, ZBM, and PGY, shows an inhibitory effect on the expression of proinflammatory cytokine interleukin IL-6 and TNF-α, which display the properties of anti-inflammatory and immune-modulating in the treatment of SLE (Fraile et al., 2012). Caffeic acid (LCW23) shared by, DS, JYH, PGY, and LQ was well-known for its pharmacological properties such as antiviral, anti-inflammatory, anti-carcinogenic, and immunomodulatory activities (Espindola et al., 2019). It has also been revealed that the administration of caffeic acid not only protect rats from cisplatin-induced oxidative stress and gastrointestinal toxicity but also reverse the activities of enzymes superoxide dismutase, catalase, glutathione reductase, and glutathione peroxidase near to their normal level, which are related to the SLE and that may be used to infer underlying mechanism of LCW on SLE (Iraz et al., 2006). Kaempferol (LCW77), a shared component of GC, HH, JYH, and LQ, was a common type of dietary flavonoid with anti-oxidative and anti-inflammatory properties. Studies also indicated that kaempferol decreased lipopolysaccharide (LPS)-induced TNF-α and IL-1 expression by increasing the number of activated macrophages, which has been reported associating with SLE (Lee et al., 2018). Additionally, quercetin (LCW97) in HH, JYH, LQ, and HL was one type of flavonoid compound with anti-cancer, anti-inflammatory and immune-regulating activities. The treatment of pristane-induced SLE model mice with liposomal quercetin found that quercetin achieves SLE therapeutic effect is by reducing the level of autoantibody expression (Dos Santos et al., 2018).

### Specific Components of Herbs in LCW

Except the shared components, most of the herbs possess their specific ingredients. For example, luteolin (LCW25), the specific component of JYH, protects against vascular inflammation in mice and TNF-α induced monocyte adhesion to endothelial cells *via* suppressing IKBα/NF-kappa B signaling pathway, which has been reported associating with SLE. Phillyrin (LCW187), one of the most effective constituents in LQ, has good antibacterial and anti-inflammatory activity, which can regulate MyD88/IκBα/NF-kappa B signaling pathway by controlling the expression of IκBα, IL-1β, IL-6 and TNF-α, which would be a benefit to SLE (Yang et al., 2017). As the major component of HL, epiberberine (LCW178) has broad biological activities, including antihyperlipidemic and antihyperglycemic effects as well as anti-inflammatory and antioxidant effects, and inhibits urease activity (Li et al., 2015) which suggested the MAPK signaling pathway could be used as the therapy target. Berberine (LCW172) was the quality marker of LCW in Chinese Pharmacopeia (China, 2015), and has anti-inflammatory effects, suppresses the expression of proinflammatory cytokines likely due to its capacity of AMPK activation, which could be used to illustrate the molecular mechanism of LCW in the therapy of SLE (Liu et al., 2019). Thus, these components could be considered as curative elements in treating SLE.

### C-T Network Construction of Active Components

**Figure 5:**
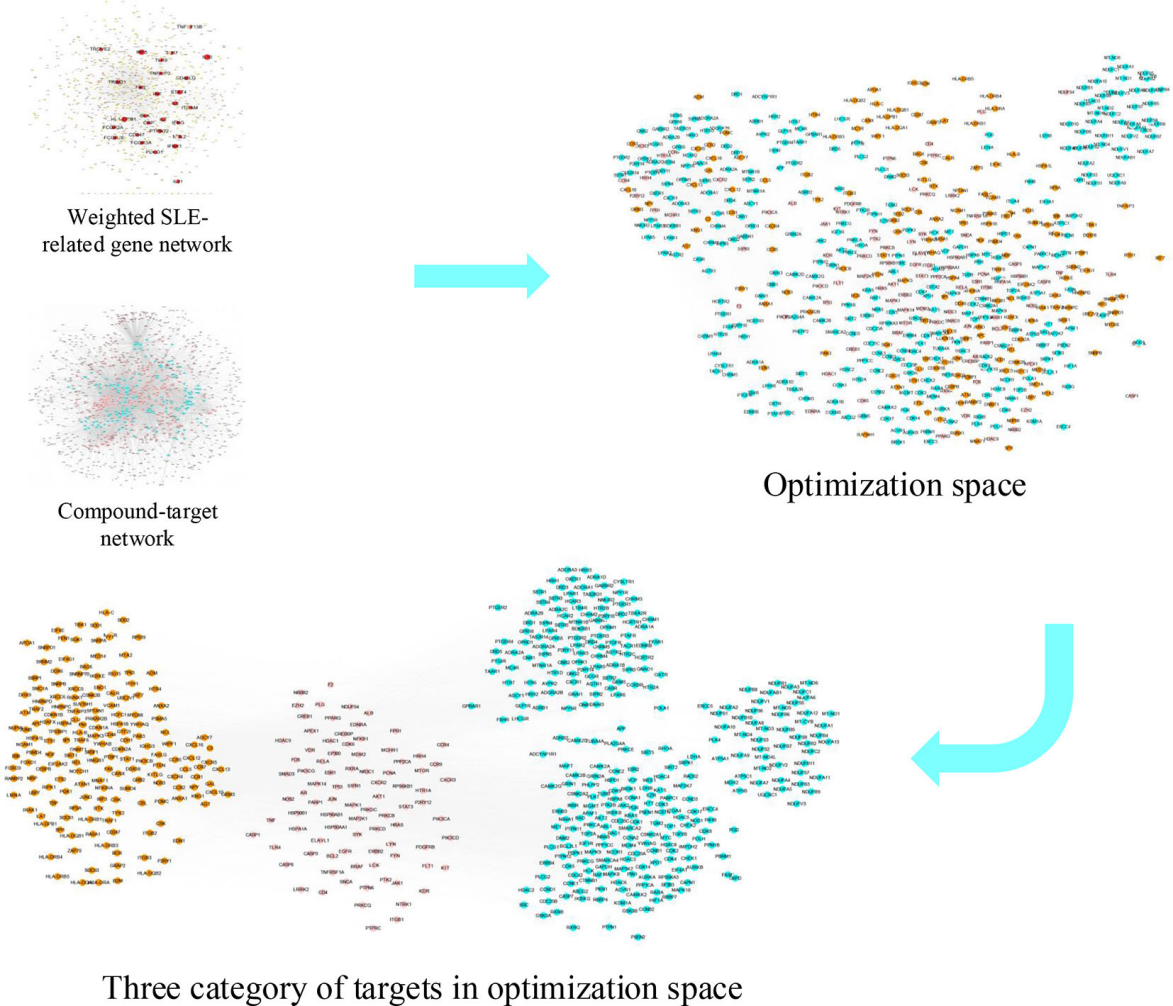
Optimization space construction. Optimization space includes three category targets, pink nodes represent essential common targets, orange nodes represent disease-specific targets, cyan nodes represent component-specific targets.

To explore the therapeutic mechanism of LCW in the treatment of SLE, 193 active components and 1,220 targets (**Table S4**) were used to construct the component-target network (**Figure 5**). This network contain 6,399 component-target associations. The average number of targets of per component is 33.16. It shows that the multi-targets characteristics of LCW for treating of SLE. Among these components, vanillic acid (LCW190, degree = 510) has the highest number of targets, followed by ferulic acid (LCW75, degree = 480), kaempferol (LCW77, degree = 300), palmitic acid (LCW100, degree = 252), luteolin (LCW25, degree = 220), protocatechuic acid (LCW29, degree = 182), beta-sitosterol (LCW5, degree = 170), stigmasterol (LCW2, degree = 170), and caffeic acid (LCW23, degree = 148). Most of these components were reported associated with the inflammation and immune-related pathways of SLE. Such as vanillic acid, a well-known flavonoid, significantly decreased the increased serum levels of TNFα and IL-6 on concanavalin a-induced liver injury in mice (Itoh et al., 2010). Moreover, it could down-regulate LPS-induced COX-2 and nitric oxide production in mouse peritoneal macrophages *in vitro* (Kim et al., 2011). Ferulic acid reduced the translocation of NF-E2-related factor 2 (NRF2) and nuclear transcription factor-κB (NF-kappa B) into the nuclei through a reduction of the expression of phosphorylated IKK and consequently inhibited IL-6 and NF-kappa B promoter activity. These data suggested that ferulic acid play anti-inflammatory roles by regulating IKK/NF-kappa B signaling pathway (Lampiasi and Montana, 2016). Protocatechuic acid inhibits Toll-like receptor-4 dependent activation of NF-kappa B by suppressing activation of the Akt, mTOR, JNK, and p38-MAPK (Nam and Lee, 2018). The function of remaining components in the treatment of SLE has been described in *Shared Components of Herbs in LCW* and *Specific Components of Herbs in LCW* sections. These results demonstrated that the crucial roles of these components in the treatment of SLE and further confirmed that these components work in a multi-target manner to treat SLE.

In the component-target network, the mean degree of targets for different components is 5.25. The top 20 targets with larger weight are ESR1, IL2, IL1B, TLR9, and ACE, etc. Interestingly, majority of these targets are related to immunity and inflammation, which are confirmed associated with the pathogenesis of SLE and that maybe indicate potential therapeutic mechanisms of LCW on SLE. For example, ESR1 polymorphism and its interaction with smoking and drinking contribute to susceptibility of SLE (Zhou et al., 2017); In addition, IL2 stimulates T cell proliferation and activation and regulates the adaptive immune response by stimulating both T-regulatory cells and activation-induced cell death in antigen-activated T cells. Some research reports indicated that IL2 region seems to play a role in the response to rituximab in SLE patients (Marquez et al., 2013); Moreover, TLR9 plays important role in immunopathology of SLE, because increased apoptosis and/or clearance deficiencies in SLE are considered to result in increased amounts of circulating plasma DNA, which may act as TLR agonists and subsequently provide B cell activation signals (Celhar et al., 2012). It is worth mention that 287-bp Alu insertion/deletion (I/D) of ACE gene was association with SLE and renal injury (Xu et al., 2007). Some other SNPs, such as A5466C, T3892C, A240T, C1237T, G2215A, A2350G, and C3409T, of ACE gene may affect the risk of certain autoimmune diseases including IgA nephropathy and lupus nephropathy (Li et al., 2010). Overall, these results suggested that LCW act synergistically to treat SLE by regulating inflammation, and immunity and further confirmed that targets of LCW were regulated by multi-components in the treatment of SLE.

### Effective Proteins Selection and Validation From Optimization Space

Here, we used the weighted gene regulatory network and active components targets network to construct disease-targets-components network. This network contains 1,829 nodes and 24,841 edges. Degree is an important topological property in the network that can be used to evaluate the importance of nodes in the network. For each node *i* in disease-targets-components network, if the degree of a node is more than the average degree of all nodes in a network, such node is believed to play a critical role in the network structure, and can be treated as a hub node (Liu et al., 2016). Following this rule, the passed nodes and their edges in the disease-targets-components network were kept and defined as optimization space. The optimization space contains 565 nodes and 15,550 edges, each node represents one effective protein, and thus we identified 565 effective proteins from the optimization space. There are three categories of effective proteins in optimization space. The first category is the direct interactions between the component targets and pathogenic genes. We defined this category as the essential common targets. The second category is the interactions of disease-specific targets. The third category is the interactions of component-specific targets (**Figure 5** and **Table S5**).

**Figure 6:**
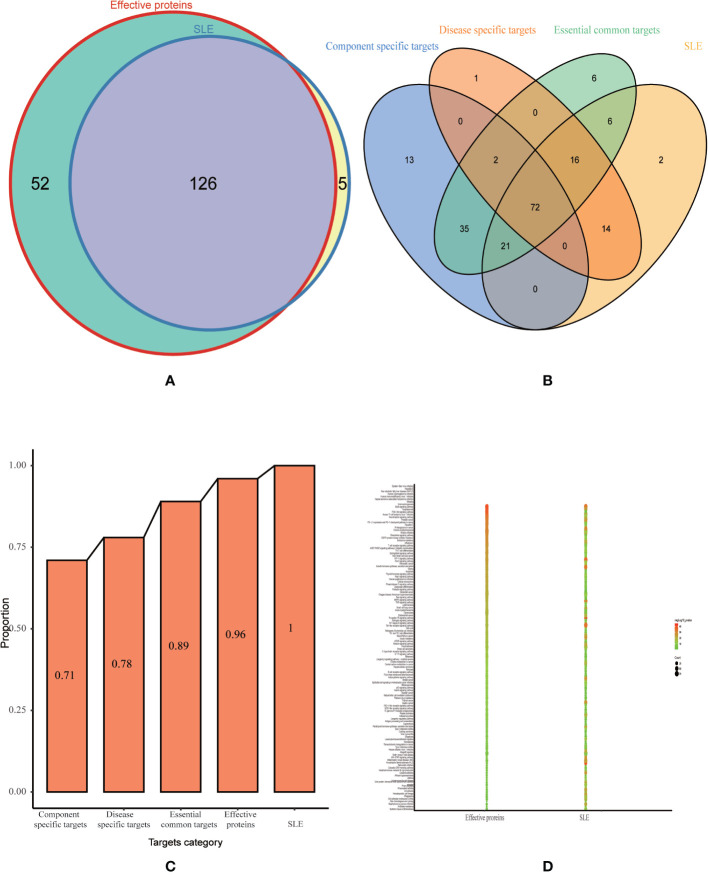
Validation of optimization space. **(A)** Venn diagram for pathway enrichment analysis of effective proteins and SLE related genes. **(B)** Venn diagram for pathway enrichment analysis of essential component-specific targets, disease-specific targets, essential common targets and SLE related genes. **(C)** The proportion histogram of enriched pathways of three categories (component-specific targets, disease-specific targets, essential common targets) and effective proteins compare with the enrichment pathways of pathogenic genes. **(D)** Bubble diagram for common pathway enrichment analysis of effective proteins and SLE related genes.

To test whether the effective proteins we selected from optimization space could cover the pathogenic genes of SLE at functional level. We performed functional pathway analysis using effective proteins and SLE pathogenic genes, respectively. Among them, the effective proteins enriched in 178 pathways (*p* < 0.05), and the pathogenic genes enriched in 131 pathways (*p* < 0.05). The effective proteins enriched pathways were found to cover 96% of the pathogenic genes enriched pathways (**Figure 6A**). Additionally, in order to test whether the effective proteins in optimization space can be replaced by essential common targets, disease-specific targets or component-specific targets for further optimization. We performed pathway analysis on essential common targets, disease-specific targets, and component-specific targets, respectively. Results show that the coverage proportion of enriched pathways of three categories compare with the enriched pathways of pathogenic genes is 89%, 78%, 71%, respectively (**Figures 6B, C**), which are far less than that of the effective proteins. These results confirmed the accuracy and reliability of our approach to construct optimization space and further demonstrated that the effective proteins selected in the optimization space play a key role in the pathogenesis of SLW.

According to the results of KEGG analysis, these effective proteins were frequently involved in PI3K-Akt signaling pathway (hsa04151), Th17 cell differentiation (hsa04659), T cell receptor signaling pathway (hsa04660), TNF signaling pathway (hsa04668), MAPK signaling pathway (hsa04010), Toll-like receptor signaling pathway (hsa04620), NF-kappa B signaling pathway (hsa04064), IL-17 signaling pathway (hsa04657), and B cell receptor signaling pathway (hsa04662) (**Figure 6D**). PI3K/Akt/mTOR signaling pathway plays an important role in cellular proliferation and growth signaling. Increased activity of Akt can reduce expression of its substrate p27kip1 in SLE (Besliu et al., 2009). This defect seems to be involved in SLE lymphocytes passage by G1/S cell cycle checkpoint. Therefore, SLE lymphocytes accumulate in S and G2/M cell cycle phases towards apoptosis or proliferation. Previous research found that abnormal activation of the PI3K/AKT signaling pathway by upregulation of CDKs and downregulation of p27Kip1 and p21WAF1/CIP1 increased the proliferation of T lymphocytes might participate in the pathogenesis of SLE in SLE patients (Tang et al., 2009). The KEGG analysis and literature reported suggested that majority of them are related to immunity and inflammation, which are confirmed associated with the pathogenesis of SLE and that may be a potential therapeutic mechanism of LCW on SLE.

### KGEC Selection and Validation

**Figure 7:**
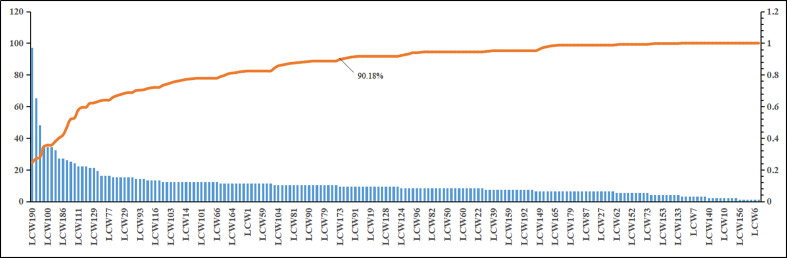
The CI and CI accumulation for KGEC selection in LCW. The bar diagram and trend line diagram are used to visualize the targets coverage of effective proteins of the LCW components and the contribution accumulation results, respectively.

The CI module was established to capture the KGEC, which would be used to illustrate the molecular mechanism of LCW in the therapy of SLE. According to the contribution accumulation results, the top 11 components including vanillic acid (LCW190), pinoresinol monomethyl ether (LCW169), phillyrin (LCW187), oleic acid (LCW160), palmitic acid (LCW100), stearic acid (LCW139), ferulic acid (LCW75), methyl caffeate (LCW137), p-coumaric acid (LCW186), ellagic acid (LCW8), and wogonin (LCW130) contribute to 51.89% target coverage of effective proteins. For further analysis, 82 components which can contribute to 90.18% targets coverage of effective proteins were selected as KGEC (**Figure 7** and **Table 3**). Among these components in KGEC, some of them belongs to different single herbs in LCW have the function of clearing away heat and toxic materials: JYH (18), LQ (19), PGY (14), HL (5), and WG (2). And some of them in different herbs have the function of cooling blood and promoting blood circulation: DH (3), CS (9), DG (4), DS (25), XS (6), TR (5), HH (21), CT (2), and ZBM (1). Higher targets coverage of effective proteins proved that the KGEC may play the leading role and generate combination effects in the treatment of SLE.

**Table 3:** The information of selected KGEC in LCW.

ID	Name	MF	MW	ID	Name	MF	MW
LCW190	Vanillic acid	C_8_H_8_O_4_	168.16	LCW43	Danshenol B	C_22_H_26_O_4_	354.48
LCW169	Pinoresinol monomethyl ether	C_21_H_24_O_6_	372.45	LCW71	Zinc14719978	C_19_H18O_4_	310.37
LCW187	Phillyrin	C_27_H_34_O_11_	534.61	LCW36	(Z)-3-[2-(E)-2-(3,4-Dihydroxyphenyl) ethenyl]-3,4-Dihydroxy-Phenyl]Acrylic Acid	C_17_H_14_O_6_	314.31
LCW160	Oleic acid	C_18_H_34_O_2_	282.52	LCW101	6-Hydroxykaempferol	C_15_H_10_O_7_	302.25
LCW100	Palmitic acid	C_16_H_32_O_2_	256.48	LCW94	Beta-carotene	C_40_H_56_	536.96
LCW139	Stearic acid	C_18_H_36_O_2_	284.54	LCW95	Cholesterol	C_27_H_46_O	386.73
LCW75	Ferulic acid	C_10_H_10_O_4_	194.2	LCW80	Licoricone	C_22_H_22_O_6_	382.44
LCW137	Methyl caffeate	C_10_H_10_O_4_	194.2	LCW66	Salviolone	C_18_H_20_O_2_	268.38
LCW186	P-coumaric acid	C_9_H_8_O_3_	164.17	LCW147	Gibberellin 7	C_19_H_22_O_5_	330.41
LCW8	Ellagic acid	C_14_H_6_O_8_	302.2	LCW175	Chrysanthemaxanthin	C_40_H_56_O_3_	584.96
LCW130	Wogonin	C_16_H_12_O_5_	284.28	LCW171	Beta-amyrin acetate	C_32_H_52_O_2_	468.84
LCW114	Mandenol	C_20_H_36_O_2_	308.56	LCW164	3-Acetyl-5-Hydroxy-7-Methoxy-2-Methylnaphthalene-1,4-Dione	C_14_H_12_O_5_	260.26
LCW111	(R)-Canadine	C_20_H_21_NO_4_	339.42	LCW131	Zinc238769177	C_21_H_24_O_6_	372.45
LCW115	Chryseriol	C_16_H_12_O_6_	300.28	LCW178	Epiberberine	C_20_H_18_NO_4_^+^	336.39
LCW138	Myristic acid	C_14_H_28_O_2_	228.42	LCW70	Zinc13341234	C_18_H_16_O_8_	360.34
LCW161	Sitogluside	C_35_H_60_O_6_	576.95	LCW1	Baicalin	C_21_H_18_O_11_	446.39
LCW129	Rutin	C_27_H_30_O_16_	610.57	LCW24	Clionasterol	C_29_H_50_O	414.79
LCW174	Cholesteryl ferulate	C_37_H_54_O_4_	562.91	LCW41	Cryptotanshinone	C_19_H_20_O_3_	296.39
LCW143	Stachyose	C_24_H_42_O_21_	666.66	LCW45	Deoxyneocryptotanshinone	C_19_H_22_O_3_	298.41
LCW118	Ethyl Linolenate	C_20_H_34_O_2_	306.54	LCW59	Nortanshinone	C_17_H_12_O_4_	280.29
LCW77	Kaempferol	C_15_H_10_O_6_	286.25	LCW106	Pyrethrin II	C_22_H_28_O_5_	372.5
LCW170	3beta-Acetyl-20,25-epoxydammarane-24alpha-ol	C_32_H_54_O_4_	502.86	LCW97	Quercetin	C_15_H_10_O_7_	302.25
LCW38	2-(4-Hydroxy-3-Methoxyphenyl)-5-(3-Hydroxypropyl)-7-Methoxy-3-Benzofurancarboxaldehyde	C_20_H_20_O_6_	356.4	LCW180	GA121-isolactone	C_23_H_32_O_5_Si	330.41
LCW185	Isoscopoletin	C_10_H_8_O_4_	192.18	LCW104	Amygdalin	C_20_H_27_NO_11_	457.48
LCW29	Protocatechuic Acid	C_7_H_6_O_4_	154.13	LCW134	Esculetin	C_9_H_6_O_4_	178.15
LCW127	(-)-Phillygenin	C_21_H_24_O_6_	372.45	LCW68	Tournefolic acid A	C_17_H_12_O_6_	312.29
LCW126	Isolariciresinol	C_20_H_24_O_6_	360.44	LCW110	Magnograndiolide	C_15_H_22_O_4_	266.37
LCW31	Tanshinone II a	C_19_H_18_O_3_	294.37	LCW81	Methoxy-7-Hydroxycoumarin	C_16_H_12_O_6_	300.28
LCW93	Shinpterocarpin	C_20_H_18_O_4_	322.38	LCW84	Glabridin	C_20_H_20_O_4_	324.4
LCW108	Quercetagetin	C_15_H_10_O_8_	318.25	LCW135	Ethyl Caffeate	C_11_H_12_O_4_	208.23
LCW113	Palmatine	C_21_H_22_NO_4_^+^	352.44	LCW55	Miltionone II	C_19_H_20_O_4_	312.39
LCW54	Miltionone I	C_19_H_20_O_4_	312.39	LCW90	Liquiritin	C_21_H_22_O_9_	418.43
LCW116	Corymbosin	C_19_H_18_O_7_	358.37	LCW3	3-Epi-Beta-Sitosterol	C_29_H_50_O	414.79
LCW25	Luteolin	C_15_H_10_O_6_	286.25	LCW123	Arctiin	C_27_H_34_O_11_	534.61
LCW9	Paeoniflorigenone	C_17_H_18_O_6_	318.35	LCW181	GA122-isolactone	C_23_H_32_O_5_Si	416.6
LCW11	Paeonol	C_9_H_10_O_3_	166.19	LCW79	Licochalcone B	C_16_H_14_O_5_	286.3
LCW103	7,8-dimethyl-1H-pyrazino[2,3-g]quinazoline-2,4-dione	C_12_H_10_N_4_O_2_	242.26	LCW188	Protocatechualdehyde	C_7_H_6_O_3_	138.13
LCW122	Bicuculline	C_20_H_17_NO_6_	367.38	LCW26	Sugiol	C_20_H_28_O_2_	300.48
LCW32	3-Hydroxymethylenetanshinquinone	C_18_H_14_O_4_	294.32	LCW34	Tanshinone II b	C_19_H_18_O_4_	310.37
LCW51	Isotanshinone IIa	C_19_H_18_O_3_	294.37	LCW173	Carthamone	C_21_H_20_O_11_	448.41
LCW14	Zinc15211904	C_17_H_18_O_6_	318.35	LCW98	Lignan	C_25_H_30_O_8_	458.55

**Figure 8:**
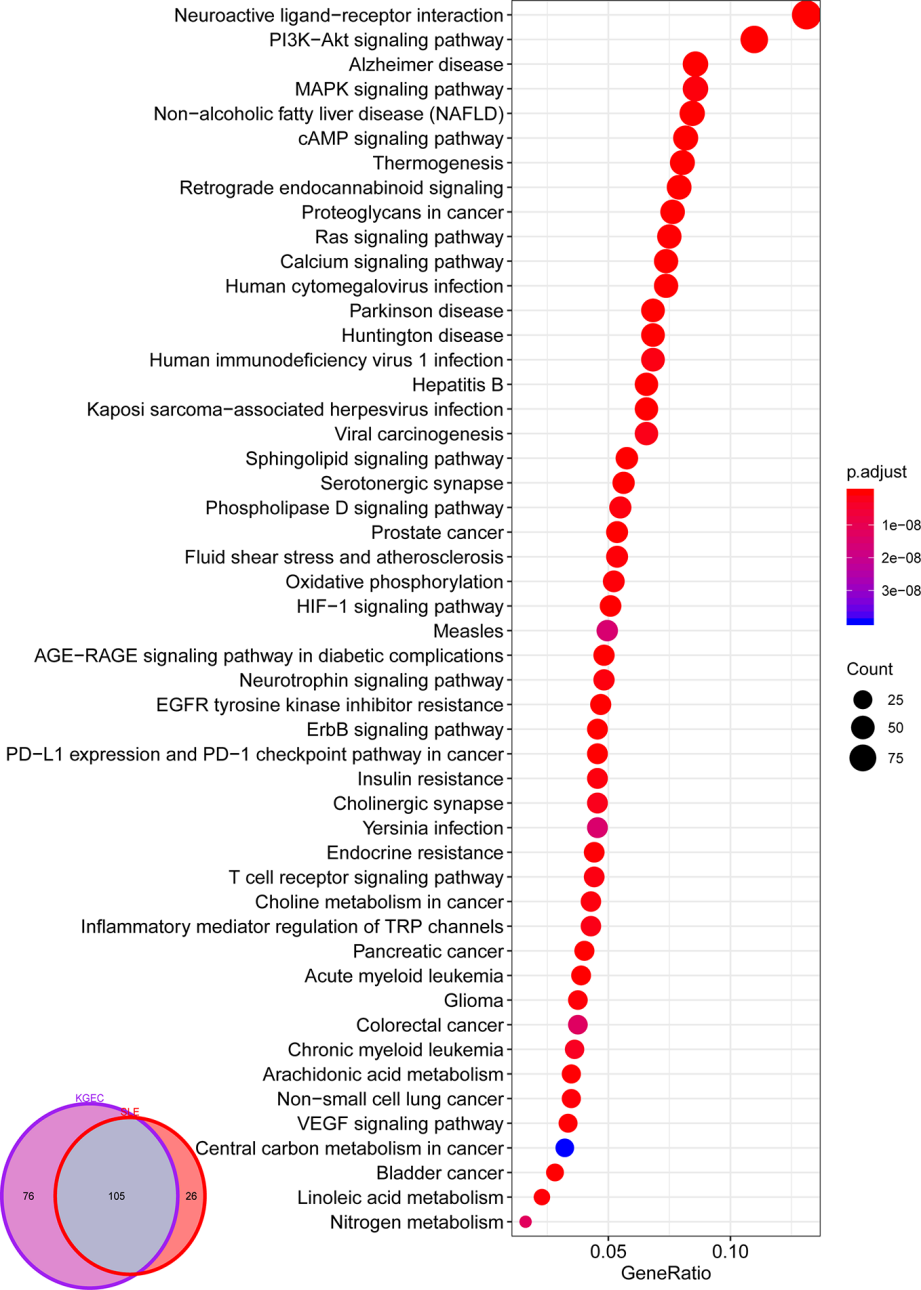
Pathway enrichment analysis of KGEC Targets. Veen diagram for pathway enrichment analysis of KGEC targets and SLE related genes; The size of the circle represents the number of genes enriched in the pathways, and the color change of the circle represents the significant degree of the enriched genes in the pathways.

In order to investigate the function of LCW in the treatment of SLE, we performed pathway analysis using KGEC targets and SLE pathogenic genes, respectively. Among them, the KGEC targets enriched in 181 pathways (*p* < 0.05), and the pathogenic genes enriched in 131 pathways (*p* < 0.05). The KGEC targets enriched pathways were found to cover 80.15% of the pathogenic genes enriched pathways (**Figure 8**). These major targets of KEGG were frequently involved in PI3K-Akt signaling pathway (hsa04151), HIF-1 signaling pathway (hsa04066), MAPK signaling pathway (hsa04010), T cell receptor signaling pathway (hsa04660), IL-17 signaling pathway (hsa04657), B cell receptor signaling pathway (hsa04662), TNF signaling pathway (hsa04668), Toll-like receptor signaling pathway (hsa04620), NF-kappa B signaling pathway (hsa04064), JAK-STAT signaling pathway (hsa04630) and Th1 and Th2 cell differentiation (hsa04658), etc. For example, the PI3K-Akt signaling pathway (hsa04151) was essential to cellular proliferation and growth. In addition, it was correlated with autoimmune diseases due to its activation in lymphocytes, which are developed features of systemic autoimmunity (Ge et al., 2017). NF-kappa B was an essential modulator in the pathogenesis of SLE in the context of the increasing immune deficiencies (Wong et al., 1999). The TLR family in the NF-kappa B pathway was responsible for sensing microbial pathogens and occupied an important position in innate immune responses. So TLR signals in B cells amplified anti-dsDNA autoantibody and enhanced one SLE characteristic, autoantibody production (Papadimitraki et al., 2006). This result suggested that the strategy of combining the optimization space with the CI model to optimize the herbal formula is reliable and the predicted KGEC may play therapy role by mediating multiple inflammation-related pathways.

### GO Enrichment Analysis of KGEC Targets

**Figure 9:**
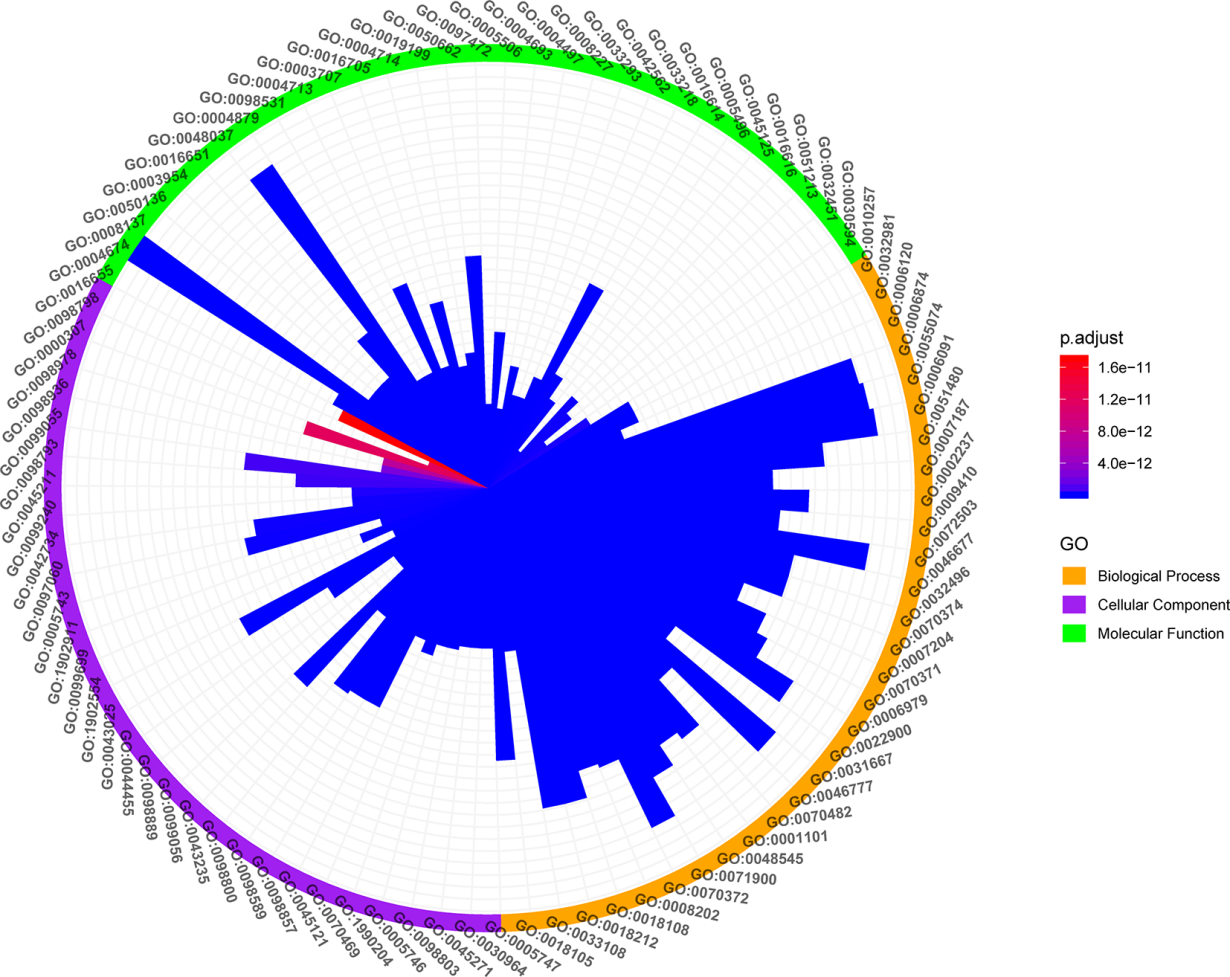
GO enrichment analysis of KGEC Targets. The Orange, purple and bright green color on the circle represent biological processes, cellular component and molecular function, respectively. The color change of the bar represents the significant degree of the enriched genes in the GO items.

GO enrichment analysis based on clusterProfiler package of R software was performed to identify the biological functional of the primary targets with *p*-values <0.05. In order to further dissect the combination effects of LCW, all the targets interacting with KGEC of LCW were enriched by GO enrichment analysis (**Figure 9**).

GO analysis showed that the targets of KGEC were enriched in biological processes related to inflammation and immunity. For example, the pathways of inflammation are regulation of inflammatory response (GO:0050727, LYN, PTGS2, PPARG, PTPRC, etc.), leukocyte activation involved in inflammatory response (GO:0002269, PTPRC, LRRK2, TNF, JUN, TLR2, etc.), production of molecular mediator involved in inflammatory response (GO:0002532, TNF, KDM6B, CXCR2, ELANE, CNR1, etc.), and inflammatory response to antigenic stimulus (GO:0002437, LYN, TLR4, F2, SERPINE1, NOS2, etc.). Important genes involved in inflammation in these pathways include TNF, NOS2, TLR2, etc. these genes are involved in inflammation of SLE (Oates et al., 2003; Marques et al., 2016; Zhao et al., 2017). The pathways of immunity are regulation of innate immune response (GO:0045088, PTPN22, LYN, PPARG, JAK1, EP300, etc.), immune response-regulating cell surface receptor signaling pathway (GO:0002768, PTPN22, BLK, LYN, PTPRC, EP300, etc.), regulation of production of molecular mediator of immune response (GO:0002700, PTPRC, IL1B, IL2, TLR9, TNF, etc.), B cell activation involved in immune response (GO:0002312, PTPRC, IL2, TLR4, LGALS1, ABL1, etc.) and T cell differentiation involved in immune response (GO:0002292, IL2, MTOR, STAT3, RORA, RORC, etc.). Important genes involved in inflammation in these pathways include PTPN22, TLR4, MTOR. These genes are involved in inflammation of SLE (Lai et al., 2015; Wong-Baeza et al., 2015; Morris et al., 2016). SLE is an autoimmune disease characterized by the presence of circulating immune complexes and inflammation in multiple organs and tissues. GO analysis confirmed that LCW treat SLE through regulation of inflammation and immune therapy.

Interestingly, LCW regulates the GO cellular component of SLE, including mitochondrial respiratory chain complex I (GO: 0005747, SNCA, NDUFS4, NDUFA4, NDUFA1, NDUFA10, etc.), mitochondrial respiratory chain (GO:0005746, SNCA, NDUFS4, NDUFA4, NDUFA1, NDUFA10, etc.), and oxidoreductase complex (GO:1990204, SNCA, NDUFS4, P4HB, NOX1, NOX4, etc.). Accumulating evidence indicates that mitochondrial dysfunction plays important roles in the pathogenesis of SLE, including mitochondrial DNA damage, mitochondrial dynamics change, abnormal mitochondrial biogenesis and energy metabolism, oxidative stress, inflammatory reactions (Lee et al., 2016). Given the accumulating evidence for mitochondrial release during inflammatory pathogenesis, these observations point to a role for mitochondria both in the stimulation of the innate immune system and as a potential source of autoantigens. Our results indicated that LCW may play a role in the treatment of SLE by modulating targets on the mitochondria.

Moreover, LCW regulates the GO molecular function of SLE including protein serine/threonine kinase activity (GO: 0004674, LRRK2, MAPK1, MTOR, EGFR, PRKCB, etc.), transcription factor activity (GO:0098531, PPARG, RXRA, ESR1, STAT3, VDR, etc.), and protein tyrosine kinase activity (GO:0004713, BLK, LYN, JAK1, EPHB2, HSP90AA1, etc.). Increasing evidence confirmed enzymes play different roles in regulating inflammation and immunity (Cunninghame Graham et al., 2007; Suarez-Fueyo et al., 2011). Our results indicate that LCW may affect different types of enzyme functions in the treatment of SLE.

### KEGG Enrichment Analysis of KGEC Targets

**Figure 10:**
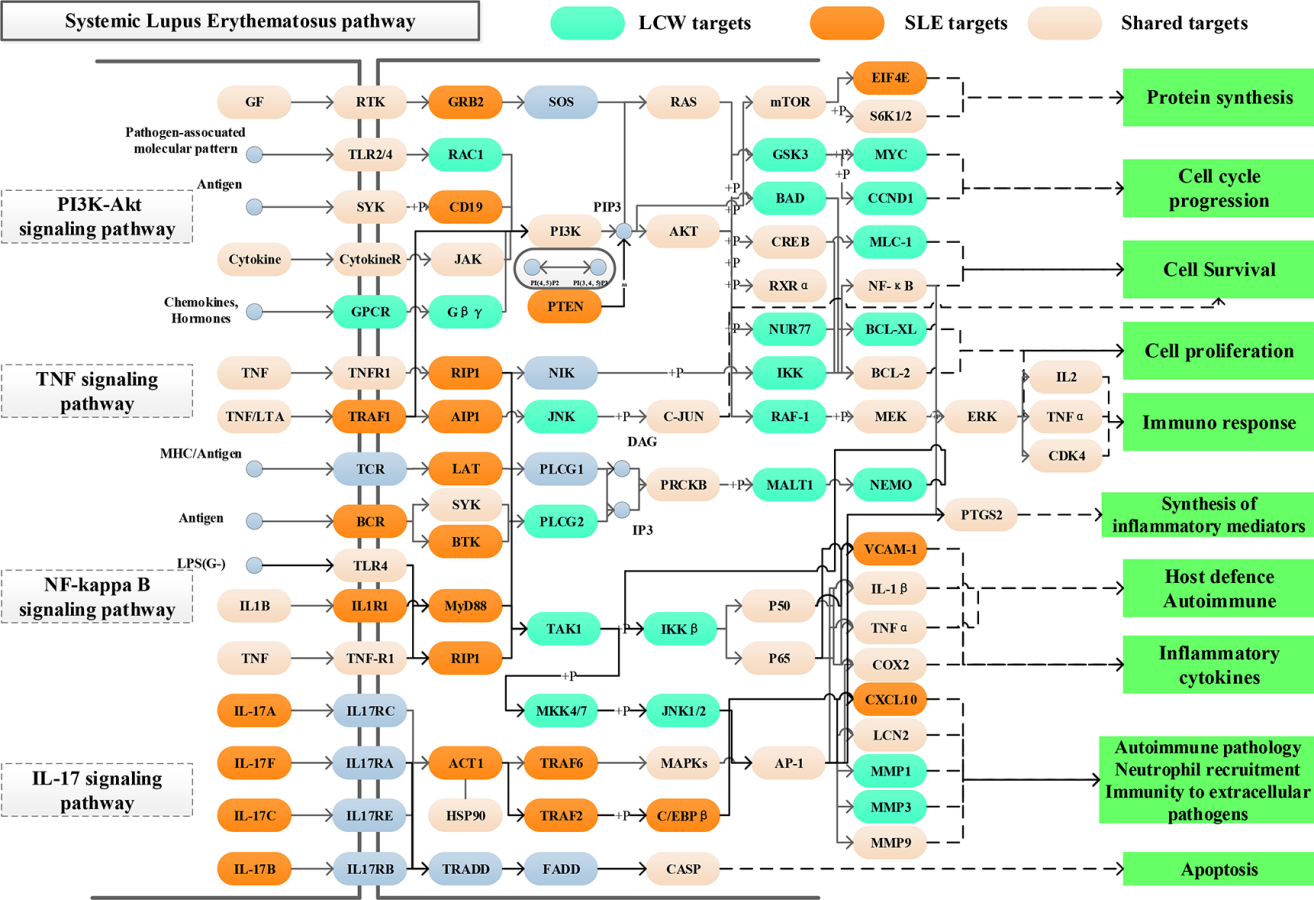
Distribution of targets of KGEC on the comprehensive signaling pathway. Different colors represent different types of targets. Light green represents the targets of LCW, orange represents the pathogenetic genes of SLE, and pink represents shared targets, respectively.

SLE is a chronic autoimmune disease involving multiple organs and systems characterized by the production of multiple autoantibodies. Previous studies confirmed that SLE-associated pathways could be decomposed into several functional modules such as immune response, synthesis of inflammatory mediators, autoimmune pathology, neutrophil recruitment, immunity to extracellular pathogens, cell cycle progression, protein synthesis, apoptosis, and so forth. Increasing evidence indicate that PI3K-Akt signaling pathway (hsa04151), TNF signaling pathway (hsa04668), NF-kappa B signaling pathway (hsa04064) and IL-17 signaling pathway (hsa04657) response to these functional modules. Such as, PI3K-Akt signaling pathway (hsa04151) has been reported involved in the inhibition of apoptosis, cell proliferation and expression of inflammatory cytokines. TNF signaling pathway (hsa04668) can induce a wide range of intracellular signal pathways including apoptosis and cell survival as well as inflammation and immunity. NF-kappa B signaling pathway (hsa04064) is the generic name of a family of transcription factors that function as dimers and regulate genes involved in immunity, inflammation and cell survival. While IL-17 signaling pathway (hsa04657) work as a subset of cytokines consisting of IL-17A-F, plays crucial roles in both acute and chronic inflammatory responses. For exploring the mechanism of LCW in the treatment of SLE at the system level, we constructed a comprehensive signaling pathway use four important molecular pathways (**Figure 10**).

These four pathways play important roles in the treatment of SLE. In order to define the position of LCW targets on the pathways, we consider the first three columns as upstream and the rest as a downstream position of the pathway. Among them, PI3K-Akt signaling pathway (hsa04151) is one of the top pathways in the treatment of SLE with KGEC in LCW. KGEC regulates 10 targets located upstream of PI3K-Akt signaling pathway (hsa04151), such as RTK, TLR2/4 and JAK, and 24 targets located downstream pathways, such as PI3K, AKT, and mTOR. The downstream targets account for more than 70%. KGEC may activate downstream of the PI3K and AKT proteins through the upstream TLR2/4, resulting in downstream GSK3, RXRα, and CREB cascade amplification, which are closely related to SLE immune response, cell proliferation and protein synthesis (Bentham et al., 2015). Most of the targets of KGEC regulating TNF signaling pathway (hsa04668) are located downstream of the pathway, such as JNK, C-JUN and RAF-1. In addition, it can also be seen (**Figure 10**) that KGEC can also affect the activation of PI3K and AKT proteins downstream of TNF to play a role in the treatment of SLE. Therefore, KGEC in LCW plays a key role in the treatment of SLE by regulating the TNF-PI3K-AKT key cascade to synergistically affect the process of immunity and inflammation.

NF-kappa B signaling pathway (hsa04064) and IL-17 signaling pathway (hsa04657) are also important pathways in the treatment of SLE by LCW. The targets regulated by KGEC are more downstream of the pathway. For example, 19 targets such as TAK1, IL-1 β, and COX2 of KGEC are located downstream of NF-kappa B signaling pathway (hsa04064). KGEC in LCW can affect upstream BCR, and then activate downstream AP-1, to further affect a series of inflammatory and immune-related proteins such as IL-1 β, TNF α, and COX2 related to SLE. The 12 targets of KGEC, such as MAPKs, AP-1 and LCN2, in the IL-17 signaling pathway (hsa04657) are located downstream of the pathway. KGEC in LCW can activate downstream AP-1, through upstream MAPKs, and affect a series of inflammatory and immune-related proteins such as LCN2, MMP1, and MMP3 related to SLE (Marques et al., 2016). Therefore, KGEC in LCW can also play a role in the treatment of SLE by regulating the NTF-MAPKs-AP-1 key cascade to synergistically affect the process of immunity and inflammation.

### Experimental Validation *In* *Vitro*

**Figure 11:**
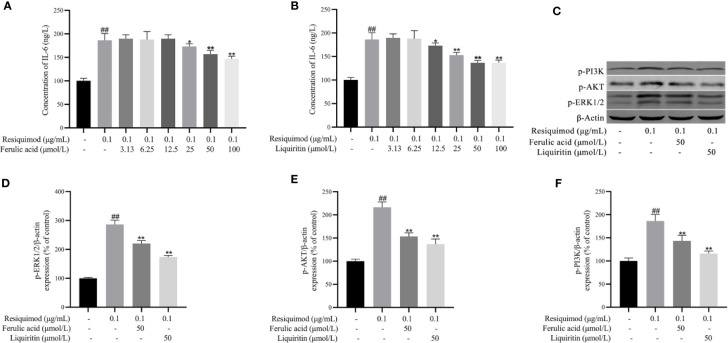
Effects of ferulic acid **(A)** and liquiritin **(B)** on resiquimod-induced RAW 264.7 cells IL-6 cytokines expression and the protein expression of p-ERK1/2, p-AKT, and p-PI3K of resiquimod induced RAW264.7 cells **(C–F)**. ^##^ *p* < 0.01, compared with control group. *p < 0.05, **p < 0.01, compared with the resiquimod group.

The effect of liquiritin and ferulic acid in KGEC was assessed in RAW 264.7 cells. As shown in **Figure S1**, 10–100 μM of liquiritin and ferulic acid revealed no obvious effects. To evaluate the anti-inflammatory effect of liquiritin and ferulic acid, the level of IL-6 was detected. Exposure of RAW 264.7 cells to resiquimod significantly elevated the level of IL-6 (*p* < 0.01). In contrast, pretreatment with liquiritin and ferulic acid significantly attenuated the phenomenon induced by resiquimod (**Figures 11A, B**). According to the anti-inflammatory effect of liquiritin and ferulic acid, 50 μM liquiritin and ferulic acid were used in subsequent research. Therefore, liquiritin and ferulic acid concentration of 50 μM were used to evaluate the effect on resiquimod-induced RAW 264.7 cells. Exposure of RAW 264.7 cells to resiquimod significantly elevated the protein expression of IL- p-ERK1/2, p-AKT, and p-PI3K. In contrast, treatment with liquiritin and ferulic acid significantly attenuated the phenomenon induced by resiquimod (**Figures 11C–F**). Our results demonstrated that liquiritin and ferulic acid inhibited the phenomenon production in resiquimod induced RAW264.7 cells.

## Discussion

The main purpose of formula optimization is to reduce the non-pharmacological factors and improve the curative effect of the formula. Although different medicinal herbs are composed as the formula according to the theory of TCM, whether the herbs or the components in the formula are necessary, especially for a specific indication is still need analysis and verification. Through optimization of formula, the medicine herbs or ingredient with effect can be screened, so that the formula is more simplified and the drug effect is more clarified.

In order to better optimize the classical formula with clinical curative effect, effective proteins, common targets, disease-specific targets, component-specific targets and KGEC targets were defined as changed target-data sets. Mathematical methods and network pharmacology were employed to investigate the coverage percentage of changed target-data sets related functional analysis on that of the disease pathogenesis genes. The changed target-data sets response to different targets of various herbs and different chemical components in each formula. To find the relatively optimal KGEC, the strategy of optimization space definition and reverse searching for components was applied and evaluated based on changed target-data sets, which provide a methodological reference for the research and development of new drugs based on TCM.

At present, how to optimize and obtain the KGEC and analyze their mechanism of action is the basis for quantification of TCM. The research of TCM emphasizes the holistic view, integrity and synergy. Network pharmacology has the characteristics of systematization and integrity, which is consistent with the philosophy of TCM research. Network pharmacology emphasizes multi-targets regulation of signal pathways to improve the therapeutic effect of drugs and reduce toxic and side effects. At present, network pharmacology is widely used in the treatment of complex diseases in TCM. For example, to determine the molecular mechanism of TCM formula in the treatment of complex diseases, such as “treating different diseases with the same treatment, treating the same diseases with different treatment”, but there are few research reports based on network pharmacology to study the optimization of formula in TCM. Thus, we propose an integrative strategy to optimize LCW based on network pharmacology, obtain the key components of LCW in the treatment of SLE, and analyze the potential mechanism of these components.

In the process of analyzing the therapeutic mechanism, network pharmacology has formed its own analytical rules, in the first step, select active components through ADME/T screening based on the chemical properties of components in TCM. Then predict targets and analyze potential mechanisms. This flowchart really solves the molecular mechanism of some formulas of TCM in treating complex diseases. Such as: Hua Yu Qiang Shen Tong Bi formula treats rheumatoid arthritis (Wang et al., 2019), Shen Qi Wan treats kidney yang deficiency syndrome (Zhang et al., 2019), and Huo Xiang Zheng Qi formula treats functional dyspepsia (Zhao et al., 2018). However, there still exists some problems, such as false positive and noise in the predicted targets of components. Here we propose a strategy to optimize active components and decode molecular mechanism of LCW in the treatment of SLE. In this strategy, we build an optimization space and extract effective proteins based on the associations of component targets and pathogenetic genes to reduce the false positive and noise.

The result shows that the enriched functional pathways of effective protein can cover 96% of the enriched functional pathways of pathogenetic genes. It proves that our strategy of constructing target-based optimization space to select effective proteins is appropriate and reliable. Based on the effective proteins provided by optimization space, we calculated the accumulation contribution degree by using the CI model, and the accumulation contribution degree of 82 active components reach more than 90% was finally optimized as KGEC. KEGG and Go analysis confirmed that the targets of our optimized KGEC are closely related to the pathogenesis of SLE in pathways and functional annotation. This proves once again the reliability of our optimization space and CI model.

Currently, our network pharmacology model provides a powerful tool for exploring the compatibility and mechanism of TCM formula. Cellular experiments were applied to prove the reliability of the network pharmacology model through verifying the protective effects of components in KGEC of LCW on the inflammation of mice RAW264.7 cells induced by resiquimod. In addition, in order to better evaluate the reliability of our proposed network pharmacology model, *in vivo* study will be conducted to verify the efficacy and mechanism of active components in the treatment of SLE in our future research.

## Data Availability Statement

The raw data supporting the conclusions of this manuscript will be made available by the authors, without undue reservation, to any qualified researcher.

## Author Contributions

A-pL, X-mQ, and D-gG provided the concept and designed the study. YG, K-xW, PW, XL, J-jC, B-yZ, and J-sT conducted the analyses and wrote the manuscript. YG, K-xW, PW, XL, J-jC, B-yZ, and J-sT participated in data analysis. A-pL, X-mQ, and D-gG contributed to revising and proof-reading the manuscript. All authors contributed to the article and approved the submitted version.

## Funding

This study is financially supported by the Startup fund from Southern Medical University [grant No. G619280010], the Natural Science Foundation Council of China [grant No. 31501080], Hong Kong Baptist University Strategic Development Fund [grant No. SDF13-1209-P01, SDF15-0324-P02(b) and SDF19-0402-P02], Hong Kong Baptist University Interdisciplinary Research Matching Scheme [grant No. RC/IRCs/17-18/04], the General Research Fund of Hong Kong Research Grants Council [grant No. 12101018, 12100719, 12102518] and the National S&T Major Project for “Major New Drugs Innovation and Development” [2017ZX09301047].

## Conflict of Interest

The authors declare that the research was conducted in the absence of any commercial or financial relationships that could be construed as a potential conflict of interest.

## Supplementary Material

Supplementary Table 1 The information of SLE genes.

Supplementary Table 2 The information of LCW components.

Supplementary Table 3 The information of LCW active components.

Supplementary Table 4 The relationship between components and targets.

Supplementary Table 5 The information of effective proteins.

Supplementary Figure 1 Effects of ferulic acid **(A)** and liquiritin **(B)** on cell viabilities.

## Abbreviations

CI, Contribution index; DL, Drug-likeness; GO, Gene Ontology; KGEC, Key group of effective components; KEGG, Kyoto Encyclopedia of Genes and Genomes; LCW, Lang Chuang Wan; OB, Oral bioavailability; PPI, Protein-protein interactions; SLE, Systemic lupus erythematosus; TCM, Traditional Chinese medicine.
