# Supplementary figures and images for: A Novel Network Pharmacology Strategy to Decode Mechanism of Lang Chuang Wan in Treating Systemic Lupus Erythematosus
Source: Front Pharmacol. 2020 Oct 2;11:512877. doi: 10.3389/fphar.2020.512877 (PMC7562735; doi:10.3389/fphar.2020.512877)

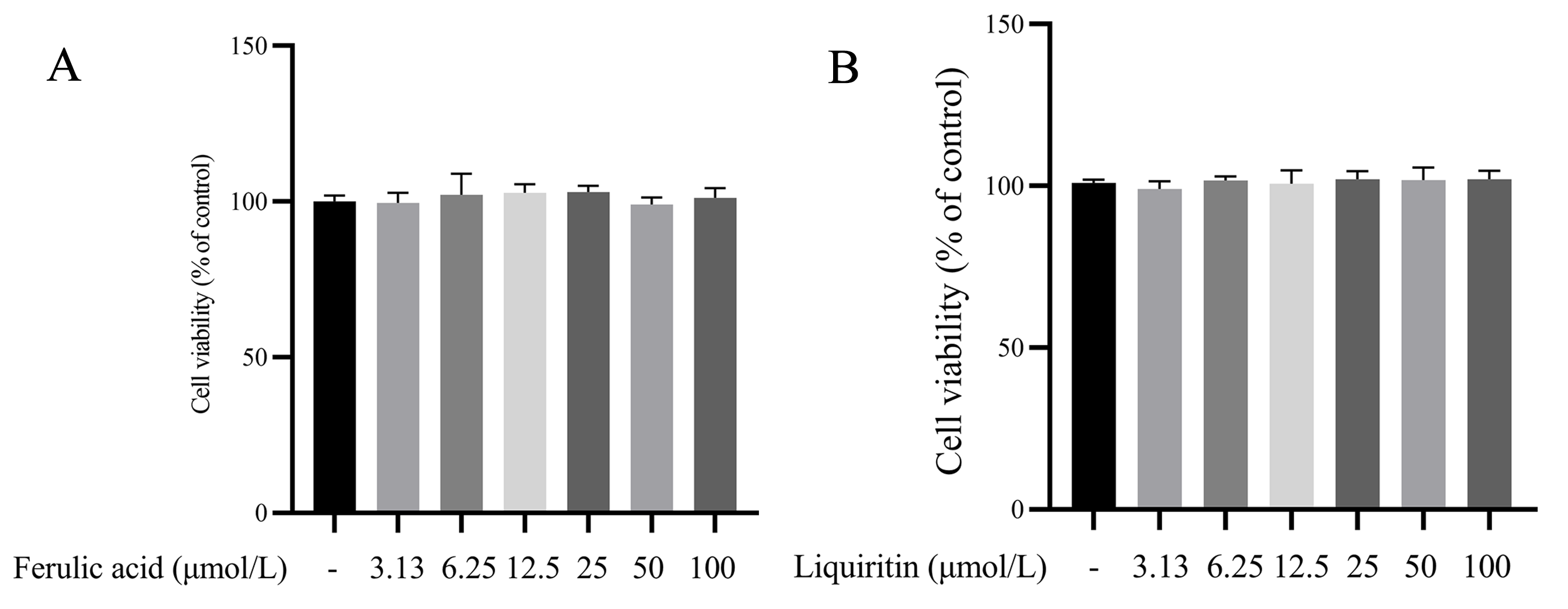

Supplement: Supplementary Figure 1 — Effects of ferulic acid (A) and liquiritin (B) on cell viabilities. [file Image_1.tif]
